# Impact on informed choice of offering antenatal sickle cell and thalassaemia screening in primary care: a randomized trial

**DOI:** 10.1258/jms.2011.010132

**Published:** 2011-06

**Authors:** Katrina Brown, Elizabeth Dormandy, Erin Reid, Martin Gulliford, Theresa Marteau

**Affiliations:** *Research Assistant*, Department of Psychology, King's College London, Health Psychology Section, 5th Floor Bermondsey Wing, Guy's Campus, London SE1 9RT; *PhD Student*, Department of Surgery & Cancer, Imperial College London; *Trial Manager*, Department of Psychology, King's College London, Health Psychology Section, 5th Floor Bermondsey Wing, Guy's Campus, London SE1 9RT; *Deputy Programme Director*, Division of Health and Social Care Research, King's College London; *Research Assistant*, Department of Psychology, King's College London, Health Psychology Section, 5th Floor Bermondsey Wing, Guy's Campus, London SE1 9RT; *Independent Researcher*; Professor of Surgery & Cancer, Imperial College London; Department of Primary Care and Public Health Sciences, King's College London, Capital House, 42 Weston St, London SE1 3QD; Professor of Health Psychology, Department of Psychology, King's College London, Health Psychology Section, 5th Floor Bermondsey Wing, Guy's Campus, London SE1 9RT

## Abstract

**Objectives:**

Offering antenatal sickle cell and thalassaemia (SCT) screening early in pregnancy can maximize the range of post-screening choices available, however these benefits should not be obtained at the expense of informed choice.  This study examined whether offering this screening in primary care at the time of pregnancy confirmation compromises women making informed choices.

**Design:**

Partial factorial, cluster randomized controlled trial.

**Setting:**

25 general practices in two socially deprived UK areas.

**Participants:**

464 pregnant women offered antenatal SCT screening.

**Intervention:**

Practices were randomly allocated to offer pregnant women screening:  i) in primary care at time of pregnancy confirmation, with parallel partner testing (*n* = 191), ii) in primary care at time of pregnancy confirmation, with sequential partner testing (*n* = 158), or iii) in secondary care by midwives, with sequential partner testing (standard care, *n* = 115).

**Main outcome:**

Informed choice – a classification based on attitudes, knowledge and test uptake.

**Results:**

91% of woman underwent screening. About a third (30.6%) made an informed choice to accept or decline screening: 34% in primary care parallel group; 23.4% in primary care sequential and 34.8% in secondary care sequential. Allowing for adjustments, rates of informed choice did not vary by intervention group: secondary care versus primary care with parallel partner testing OR 1.07 (95% CI 0.56 to 2.02); secondary care versus primary care with sequential partner testing OR 0.67 (95% CI 0.36 to 1.25).  Uninformed choices were generally attributable to poor knowledge (65%).

**Conclusion:**

Offering antenatal SCT screening in primary care did not reduce the likelihood that women made informed choices. Rates of informed choice were low and could be increased by improving knowledge.

## INTRODUCTION

There is a consensus that choices made about undergoing antenatal screening for fetal abnormalities should be informed.^1–3^ Research on the factors associated with informed choice for people making healthcare choices has tended to assess one dimension only, most often knowledge about a procedure. It is now widely acknowledged that informed choice is more complex than this and involves several dimensions. A consensus is emerging that informed choices have two core characteristics: first, they reflect an individual's values, and second, they are made in the context of good knowledge. An informed choice has been defined as:

*A decision based on relevant knowledge, consistent with the decision-maker's values and behaviourally implemented*.^4^

Thus, an informed choice to accept screening is one based on good knowledge where those with positive attitudes towards undergoing screening accept it; an informed choice to decline screening is one based on good knowledge where those with negative attitudes towards undergoing screening decline it. Choices based on poor knowledge or which are inconsistent with attitudes towards undergoing the screening test are classified as uninformed.

Many factors have been identified as important in limiting the opportunity for couples to make informed choices in the context of antenatal screening for sickle cell and thalassaemia (SCT). These include: failure to offer screening or screening offered too late in pregnancy; women's lack of knowledge about the screening test; the screening process taking too long; and diagnostic testing offered too late in pregnancy.^5,6^ In 2001 a national antenatal screening programme for sickle cell and thalassaemia in England was set up. Pregnant women are informed about the screening tests by their midwife, and this discussion is supported by written materials produced nationally (http://sct.screening.nhs.uk/publications). In the study sites a universal screening policy was in place. Thus all women were offered screening for both sickle cell and thalassaemia using a single blood sample (testing for routine red cell indices and haemoglobin variants allows identification of both disorders).

Offering screening early in pregnancy has the potential to maximize early uptake and the range of reproductive choices, including prenatal diagnostic testing (PND) for at-risk couples (where both parents are carriers of the condition), and the choice of termination of affected pregnancies. The target is to offer screening by 8–10 weeks gestation, but this rarely happens.^2,7^ The earliest stage at which antenatal screening can be offered, practically, is at the pregnancy confirmation consultation in primary care. However, some characteristics of GPs' primary care consultations may impede the facilitation of informed choices. The duration and context of the primary care pregnancy confirmation consultation is perhaps less conducive to facilitating informed choices than is the booking appointment conducted by a midwife. GPs have less time than midwives for their first consultation with a pregnant woman. Further, women booking with a midwife may have had their pregnancies confirmed several weeks earlier, and are therefore more likely than women just confirming their pregnancies to be primed to receive, retain and process information regarding antenatal care. This study examines whether offering antenatal SCT screening in primary care at the time of pregnancy confirmation compromises the making of informed choices. This is part of a trial examining the effectiveness, acceptability and feasibility of offering screening in primary care. The trial took place in areas with high prevalence of sickle diseases and thalassaemia, due to their large black and minority ethnicity communities. We report separately that offering screening in primary care results in screening taking place earlier in pregnancy than when screening is offered by midwives in community-based secondary care.^7^

## METHODS

### Design

A partial factorial cluster randomized controlled trial design was used to assess rates of informed choice in women offered antenatal SCT screening.^7^

### Setting and randomization

The study was conducted in 25 practices from two UK inner-city PCTs, ranked sixth and thirteenth most deprived of the 354 PCTs in England,^8^ with about 40% of their populations from minority ethnic groups. Antenatal SCT screening was offered to all women regardless of ethnicity during the study period.^9^ The standard pattern of care at the time of the study was for women to be offered screening at their first midwife appointment (at about 12–15 weeks gestation).

Practices were allocated to one of three study groups:

*Primary Care Parallel:* Screening offered to the woman and her baby's father simultaneously at the first consultation to confirm pregnancy in primary care (analogous to couple screening in antenatal screening for cystic fibrosis). If the baby's father did not attend with the mother at the first consultation, then she received a testing information pack to give to him.

*Primary Care Sequential:* Screening offered to the woman at the first consultation to confirm pregnancy in primary care, screening subsequently offered to the baby's father only if the woman is identified as a carrier.

*Secondary Care Sequential:* Screening offered to the woman at her first midwife appointment (booking appointment) in community-based secondary care, screening subsequently offered to the baby's father only if the woman is identified as a carrier.

SCT screening involves testing a blood sample from the parent. Women wishing to be screened had this sample taken in accordance with the procedures operating where the test was offered. In some practices an in-house phlebotomy service was available (*n* = 10) and in others women were required to attend a local phlebotomy centre (*n* = 15). Health-care professionals were trained to facilitate informed choice about SCT screening, through a teaching session on key points to be communicated to the patient, a chance to practice what they had learned in a simulated consultation with an actor playing the patient, and a question-and-answer session with a local Sickle Cell counsellor.^10^ This discussion was supported by written materials produced nationally by the National Screening Committee and NHS Sickle Cell and Thalassaemia Screening Programme (http://sct.screening.nhs.uk/publications). The study information leaflet did not provide any additional information on the screening test itself.

### Randomization

Study practices were allocated to intervention groups after they had agreed to participate and entered the run-in data collection period (when data on pre-trial gestational age at screening were collected). The allocations for 27 practices (two practices withdrew from the study before the intervention started) were determined independently by the trial statistician, in three batches, using minimization;^11^ stratifying for the two primary care organizations and number of family physician partners at the practice (one or two versus three or more).

### Participants

Women eligible to complete assessments of informed choice comprised those who attended a participating practice to report their pregnancies and:

– planned to continue their pregnancies
– were less than or equal to 19 weeks and six days gestation at their consultation to confirm pregnancy in primary care (based on self-reported last menstrual period)
– for whom there was no written record of sickle cell and thalassaemia carrier status in primary care
– were aged 18 or over
– agreed to be contacted by the research team.

Women who miscarried before being contacted by the research team were also excluded.

### Response rate

There were 993 eligible women who agreed to be contacted by the research team. Of these women, 727 (73%) agreed to take part and 511 (70%) completed questionnaires were received. Completed questionnaires were obtained from 464 (68%) women who met the eligibility criteria for the main analysis.

### Outcome measures

The questionnaires used in this study (see Appendix 1) were developed for use in populations with low levels of literacy.^12^

*Knowledge:* This 10-item scale (questions 5–16 in Appendix 1) assessed knowledge in the main areas deemed important in professional guidelines for informed consent for screening,^13^ made specific to screening for sickle cell and thalassaemia. The alpha coefficient of internal reliability in this sample was 0.66. Non-responses for knowledge were coded as incorrect. Good and poor knowledge were defined by the midpoint of the scale, with scores greater than five denoting good knowledge. There is no absolute standard of good knowledge – it could be argued that only answering all questions correctly constitutes good knowledge. The decision to use scores greater than five is a pragmatic one.

*Attitudes towards undergoing the test:* This scale consisted of four items (items 1–4 in Appendix 1, each scored 0 to 6, scale score range 0 to 24), assessing the extent to which women perceive undergoing SCT screening themselves (not SCT screening *per se*) positively or negatively. The alpha coefficient of internal reliability in this sample was 0.70. As there were four questions, the scale mid-point represents the split between positive and negative responses; positive and negative attitudes were therefore defined by the midpoint of the scale, with scores greater than 11 denoting positive attitudes towards undergoing screening and scores of 11 or less denoting negative attitudes towards undergoing screening.

*Screening uptake:* This was extracted from laboratory records.

*Informed choice:* Choices about antenatal SCT screening were classified as informed or uninformed according to women's knowledge about antenatal SCT screening, their attitudes towards undergoing screening and whether or not they underwent screening. Women with positive attitudes towards undergoing screening (attitude score greater than 11) and good knowledge (knowledge score greater than five) who underwent antenatal SCT screening, were classified as making an informed choice to undergo screening. Women with negative attitudes and good knowledge who did not have screening, were classified as making an informed choice to decline screening. Other choices were classified as uninformed. There is good evidence to support the validity of this classification.^4,12^ Total rates of informed choice were calculated by summing the number of women who made an informed choice to accept screening with the number who made an informed choice to decline the screening test.

### Demographic details

Participants provided their date of birth, gestation at time of questionnaire completion and highest level of education. Information on parity and ethnicity was obtained from GP practices. Neighbourhood levels of deprivation data were estimated from post codes to which study materials were sent.^14^

### Procedure

Ethical approval was granted for the trial (05/Q0501/36). Women were verbally informed of the study and provided with a study information leaflet (in their own language) by the attending health-care professional at their first visits to confirm their pregnancy in primary care. Women were asked to verbally consent to their contact details being given to the research team. A female researcher contacted those agreeing to this, by telephone in the first instance, to seek informed consent to participate. Consenting women were invited to complete questionnaires on the telephone or by post. Up to two reminders were sent together with a questionnaire. Women with a preferred language other than English were invited to complete the questionnaire in the language of their choice. Twenty languages other than English were used in telephone translations and nine languages other than English were used in written translations. About half of the women completed the questionnaire after they had provided a blood sample for the screening test.

A choice of telephone or postal completion was offered in order to achieve higher response rates. Telephone completion compared with postal completion allows for ready translation to more languages and provides social support to complete the questionnaire.^15^ Perhaps most importantly it removes the reading obstacle to questionnaire completion thereby allowing the estimated 20–25% of the UK population who are functionally illiterate^16^ to participate in the research process.

### Data analysis

Proportions and medians (with interquartile ranges), for attitude and knowledge scores and for informed choice, were tabulated by study group. Influence of study group on each of these variables was assessed using logistic (non-linear outcome variable) or linear regression (linear outcome variable) of the variable on study group using robust standard errors to allow for clustering by practice. Logistic regression was used to estimate odds ratios and their confidence intervals for making an informed choice by study group. Multiple linear regression was used to identify independent predictors of knowledge. In both analyses, adjustment was made for age group, parity, ethnicity, education, Indices of Multiple Deprivation (IMD) score, language (English or translated) and method of questionnaire completion.

## RESULTS

### Characteristics of the sample

Table 1 summarizes the demographic characteristics of women in each of the three trial arms. The groups did not differ on parity, IMD 2004 score, educational level, age, method of completion or use of translated questionnaires. Significant differences were observed in ethnicity and the gestation at which questionnaires were completed. For the Primary Care Sequential group, 48.7% were described as Asian, in comparison with 22% in Primary Care Parallel and 28.7% in Secondary Care Sequential. For the Primary Care Parallel group, 34.6% were African/African Caribbean ethnicity, in comparison with 15.2% in Primary Care Sequential and 21.7% in Secondary Care Sequential. The gestation at completion of the questionnaires was eight weeks (7–11) for Primary Care Parallel, nine weeks (8–12) for Primary Care Sequential, and 18 weeks (16–21) for Secondary Care Sequential.

**Table 1:** Demographic characteristics

	Primary Care Parallel (*n* = 191)	Primary Care Sequential (*n* = 158)	Secondary Care Sequential (*n* = 115)^a^	*P* value for between- group comparison
Primiparae^†^	61.3	57.0	58.3	0.83
IMD score^‡^	39.7 (32.3 to 49.2)	35.9 (28.7 to 41.7)	36.2 (31.4 to 43.7)	0.11
Highest educational qualification^†^				
No qualification	7.9	8.9	9.6	0.06
GCSE or similar	15.7	20.9	22.6	
GCE A-level or similar	14.1	16.5	7.0	
Further education or similar	20.9	17.1	20.9	
Degree or similar	38.7	35.4	37.4	
Missing	2.6	1.3	2.6	
Age at completion of questionnaire (years)^‡^	28.2 (24.2 to 31.3)	28.4 (25.9 to 33.2)	30.0 (25.0 to 33.6)	0.173
Gestation at completion of questionnaire (weeks)^‡^	8 (7 to 11)	9 (8 to 12)	18 (16 to 21)	<0.001***
Practice-reported ethnic group^†^				
Asian	22.0	48.7	28.7	<0.001***
African/African Caribbean	34.6	15.2	21.7	
North European	10.0	6.3	13.9	
South European	5.2	3.8	8.7	
Other	24.1	20.9	21.7	
Mixed	2.6	2.5	3.5	
Not recorded	1.6	1.3	0.9	
Other non-North European	0.0	1.3	0.9	
Questionnaire completed on telephone^†^	88.0	84.2	83.5	0.71
Questionnaire translated^†^	16.2	27.2	17.4	0.24

^a^one case had missing values for informed choice and attitude; ^†^%; ^‡^median (interquartile range); ***P < 0.001

### Rates of informed choice and method of test offer

Less than a third of women made an informed choice to accept or decline screening (30.6%) (Table 2). Attitudes towards undergoing antenatal SCT screening were strongly positive and did not vary by trial arm (*P* = 0.55). The proportion of women with positive attitudes was 96.3% for Primary Care parallel, 94.3% for Primary Care Sequential and 96.5% for Secondary Care Sequential. The mean difference between the Primary Care Sequential and Primary Care Parallel arms was −0.20 (95% confidence interval: −.65 to 0.45, P = 0.708). The mean difference between the Secondary Care Sequential and Primary Care Parallel arms was −0.33 (−0.41 to 1.07, *P* = 0.367). The majority of women acted consistently with their positive attitudes towards undergoing screening in that they underwent screening. Knowledge was low and did not vary by trial arm. Screening uptake did not vary by trial arm. The relative odds of screening uptake in the Primary Care Sequential arm, compared with the Primary Care Parallel arm, were 0.81 (95% confidence interval 0.36 to 1.82, *P* = 0.616); the relative odds for the Secondary Care Sequential arm, compared with Primary Care Parallel, were 0.53 (0.20 to 1.42, *P* = 0.205). Rates of informed choice did not vary by trial arm. The relative odds of an informed choice in the Primary Care Sequential arm, compared with the Primary Care Parallel arm, were 0.59 (0.26 to 1.36, *P* = 0.219); the relative odds for the Secondary Care Sequential arm, compared with Primary Care Parallel, were 1.03 (0.49 to 2.17, *P* = 0.930). The majority of choices were classified as uninformed because of poor knowledge (65.3%), and not because woman acted inconsistently with their attitudes (4.1%). Women knew most about options following antenatal diagnosis and sickle cell disease, and least about thalassaemia and inheritance of the conditions.

**Table 2:** Attitudes, knowledge, screening uptake and informed choice among women

	Primary Care Parallel (*n* = 191)	Primary Care Sequential (*n* = 158)	Secondary Care Sequential (*n* = 115)^a^	All (*n* = 464)	*P* value for between-group comparison
Making an informed choice^†^	34.0	23.4	34.8	30.6	0.38
Uninformed choice, poor knowledge^†^	62.8	72.1	60.0	65.3	0.47
Uninformed choice, attitude-behaviour inconsistent^†^	3.1	4.4	5.2	4.1	0.75
Uptake^†^	92.7	91.1	87.0	90.7	0.45
Attitude median score					
(0–24, higher score = more positive attitude)^‡^	23 (20–24)	23 (19–24)	24 (20–24)	23 (20–24)	0.55
Proportion with positive attitudes^†^	96.3	94.3	96.5	95.7	0.60
Proportion acting consistently with attitudes: POSITIVE attitude, TESTED^† ††^	93.5 (172/184)	93.3 (139/149)	88.3 (98/111)	92.1 (409/444)	0.44
Proportion acting consistently with attitudes: NEGATIVE attitude, NOT TESTED^† ††^	29 (2/7)	44 (4/9)	50 (2/4)	40 (8/20)	0.81
Knowledge median score					
(0–10, higher score = better knowledge)^‡^	5 (3–6)	4 (2–6)	5 (3–6)	4 (3–6)	0.33
Proportion with good knowledge^†^	37.2	27.9	40.0	34.7	0.47

^a^one case had missing values for informed choice and attitude; ^†^%; ^††^n/N; ^‡^median (interquartile range)

### Predictors of making an informed choice

Education, age, language of questionnaire completion and ethnicity were all significant predictors of making an informed choice (Table 3). Failure to disclose education (OR 11.39, 95% CI 1.62 to 80.2), having a degree (OR 7.30, 95% CI 1.37 to 38.9), or being educated to GCSE/A-level/Further education (OR 6.26, 95% CI 1.25 to 31.3) versus no education, were the strongest predictors of making an informed choice. Being in the oldest age category (OR 2.84, 95% CI 1.23 to 6.57) was the next strongest predictor. Having the questionnaire translated (OR 0.13, 95% CI 0.05 to 0.36) or being from a high-risk ethnic group (OR 0.50, 95% CI 0.26 to 0.93) predicted an uninformed choice.

**Table 3:** Predictors of women making an informed choice

		Relative odds of making an informed choice (OR)	95% C.I.	*P* Value
Age (years)	<24	1.00	–	
	24–27.9	1.13	(0.46 to 2.77)	0.793
	28–31.9	2.02	(0.96 to 4.25)	0.063
	≥32	2.84	(1.23 to 6.57)	0.014*
	Not known	1.04	(0.16 to 6.84)	0.971
Parity	Multiparous	1.00	–	
	Primiparous	1.30	(0.81 to 2.10)	0.282
Ethnicity	Low risk	1.00	–	
	High risk	0.50	(0.26 to 0.93)	0.030*
Education	None	1.00	–	
	GCSE/A-level/Further Ed.	6.26	(1.25 to 31.3)	0.025*
	Degree or above	7.30	(1.37 to 38.9)	0.020*
	Not known	11.39	(1.62 to 80.2)	0.015*
IMD 2004 score		0.99	(0.97 to 1.02)	0.6481
QR language	English	1.00	–	
	Translated	0.13	(0.05 to 0.36)	<0.001***
Completion method	Postal	1.00	–	
	Telephone	2.03	(0.93 to 4.44)	0.077
Study group	Secondary Care Sequential	1.00	–	
	Primary Care Parallel	1.07	(0.56 to 2.02)	0.843
	Primary Care Sequential	0.67	(0.36 to 1.25)	0.212

*P < 0.05; ***P < 0.001

### Predictors of good knowledge

Older age, more education and telephone questionnaire completion all predicted better knowledge. Having the questionnaire translated predicted poorer knowledge (Table 4).

**Table 4:** Predictors of women's knowledge

		Relative odds of having good knowledge (OR)	95% C.I.	*P* Value
Age (years)	<24	0		
	24–27.9	0.32	(−0.33 to 0.96)	0.318
	28–31.9	0.83	(0.19 to 1.47)	0.013*
	≥32	0.94	(0.23 to 1.64)	0.011*
	Not known	−0.09	(−1.87 to 1.70)	0.919
Parity	Multiparous	0		
	Primiparous	−0.12	(−0.27 to 0.51)	0.529
Risk based on ethnicity	Low SCT risk	0	–	
	High SCT risk	−0.51	(−1.03 to 0.04)	0.051
Highest educational qualification	No qualification	0		
	GCSE/A-level/Further Education	0.66	(−0.11 to 1.42)	0.087
	Degree or above	1.36	(0.54 to 2.18)	0.002**
	Not known	0.90	(−0.21 to 2.01)	0.133
IMD 2004 score		−0.02	(−0.04 to 0.01)	0.114
QR language	English	0		
	Translated	−2.10	(−2.65 to −1.56)	0.001**
Completion method	Postal	0		
	Telephone	1.07	(0.43 to 1.71)	0.002**
Study group	Secondary Care Sequential	0		
	Primary Care Parallel	0.11	(−0.43 to 0.66)	0.667
	Primary Care Sequential	−0.35	(−0.81 to 0.10)	0.120

*P < 0.05; **P < 0.01

## DISCUSSION

Being offered antenatal SCT screening in primary care at the time of pregnancy confirmation did not undermine women making informed choices. Women were as likely to make an informed choice when the test was offered in primary care as when it was offered by midwives later in pregnancy. However, less than one-third of women made an informed choice about screening. This is much lower than has been observed in the context of other antenatal screening tests.^17–19^ This may be because among health-care professionals, pregnant women and their families awareness of sickle cell and thalassaemia, and of screening for these conditions, is lower than awareness of Down syndrome.

Almost all uninformed choices were classified as such because of poor knowledge, rather than because of inconsistency between attitudes and behaviour. The proportion of women with good knowledge in the present study was also lower than that observed in the context of Down syndrome screening.^17–19^ This may be because levels of neighbourhood deprivation were greater in the current sample or because greater effort was made to include people who did not speak English and are often excluded from the research process. Alternatively, knowledge of sickle cell and thalassaemia may be poorer than knowledge of Down syndrome. Asking the baby's father to be tested (parallel partner testing arm – Group 1) was not associated with better knowledge, a somewhat surprising result given tests for fathers during pregnancy are uncommon and so this unusual request was expected to prompt better information provision from health-care professionals or more questioning from mothers and partners. The measure of informed choice categorized good and bad knowledge as above or below the scale midpoint (which was also the sample median). If the cut-off for good knowledge was higher, even fewer women would have made an informed choice.

Levels of informed choice in this sample did not appear to be undermined by attitude-behaviour inconsistency, that is, the great majority of women who had a positive attitude towards undergoing testing did so. Scores above the scale midpoint were defined as positive attitudes because a marked positive skew in attitudes meant very few participants would fall into the negative category were a median split applied. The findings must therefore be interpreted in light of this analytic decision.

Being educated to degree level, being older and being in a low-risk ethnic group were all significant predictors of making an informed choice and (with the exception of ethnicity) of having good knowledge. Patient characteristics have been shown to predict the amount and type of information provided by health-care professionals, with patients of lower socioeconomic status,^20^ younger patients,^21,22^ and patients from minority ethnic groups^23^ being less likely to receive adequate information. It is not possible to ascertain from the current study whether less educated, younger women from minority ethnic groups were less likely to make informed choices because of limited information provision at the point of screening offer, or because of the way in which those women went on to process that information.

The effect of method of questionnaire completion on knowledge may be explained by the greater tendency among postal responders than telephone responders to answer ‘don't know’, observed in this sample and elsewhere.^15,24^

There is evidence that whilst women will accept professional advice about screening, they are very keen to be offered a choice and to be given information about antenatal screening to inform that choice.^25–27^ It may be that informed choice in the context of sickle cell and thalassaemia screening would be achieved if the screening test were offered pre-conceptually (by screening for haemoglobin variants before a pregnancy occurs, as is routine practice in some countries).^10,28,29^ After adjustment there was no difference in rates of informed choices across the three trial arms. There is therefore no evidence from this trial that offering the test in primary or secondary care results in differing rates of informed choices. However, offering the test in primary care increases the proportion of women screened by 8–10 weeks.^7^

### Strengths and limitations

This study assessed knowledge and attitudes towards undergoing antenatal SCT screening in a socioeconomically, ethnically and linguistically diverse sample. The study sample is representative of populations in geographical areas where antenatal SCT screening is offered routinely to all pregnant women, and, as such, provides an ecologically valid estimate of the impact that screening in primary care may have on rates of informed choice. The suboptimal (though above average for a study of this population) questionnaire response rate of 66% may have biased our results. Response rates were similar amongst randomization groups, so this is unlikely to have biased our main comparison. Given that non-responders are likely to have poorer knowledge than responders, we have probably overestimated rates of informed choice.

The study was designed to allow an estimate of the proportions making an informed choice in each group within 10%, with 95% confidence, allowing for clustering. However, the achieved sample size was smaller than anticipated, while the intraclass correlation coefficient by general practice for the measure of informed choice was higher than expected (0.089). We acknowledge that the study had limited power to detect modest differences that might have been of clinical importance. Whilst the point estimates suggest a slightly lower proportion making informed choices in the primary care sequential group, this may have been a chance finding as the confidence intervals were wide and there was no appreciable evidence against the null hypothesis. Although it was not feasible to extend this study, we have no reason to expect that, were the sample size increased, primary care sequential testing would emerge as significantly worse for informed choice than either primary care parallel testing or secondary care sequential testing.

Training was provided to health-care professionals in facilitating informed choice,^10^ but the quality of communication between health-care professionals and pregnant women was not independently assessed. Alongside the impact of training, health-care professionals' attitudes to screening may affect whether their patients make informed choices,^30,31^ however the evidence is mixed and mainly in relation to antenatal Down syndrome screening. More research is required to explore the relationship between health professional training, attitudes and facilitation of informed choice in the context of haemoglobinopathies.

This study does not assess the effect of making an informed choice on outcomes such as prenatal diagnosis (PND) uptake or termination uptake. We estimate an adequately powered study to answer this question would require a sample size equivalent to the pregnant population of England for one year, and as such was not feasible as part of this trial.

### Implications for practice

Whilst most women in this sample were keen and able to undergo antenatal SCT screening, a sizeable proportion did so in the context of poor knowledge. Poor knowledge undermines informed choices and increases the likelihood of anxiety in those who are identified as carriers.^32,33,34^ While providing written information about screening tests increases knowledge,^29^ increasing knowledge in those with low levels of education is more effectively achieved if those providing information also check understanding, and clarify areas not understood.^35^ Decision aids which help parents to consider their values and preferences whilst also providing information may be effective in this context.^36^

### Conclusion

Offering antenatal SCT screening in primary care, at the time of pregnancy confirmation, did not compromise women making informed choices. Rates of informed choice were low for all participants. Efforts to improve rates of informed choice should focus on improving knowledge, particularly in those with low levels of education and whose first language is not English.

## Trial registration

Registered with International Standard Randomized Controlled Trial Number Register (ISRCTN00677850).

## Funding

This research was funded by a grant from the NIHR Health Technology Assessment Programme (UK) (Reference number: NHS HTA 03/02/03; Title: Antenatal screening for haemoglobinopathies in primary care: a cluster randomized trial to inform a simulation model; Principal Investigator: TMM). The funder approved but had no role in the design of the study, in collection, analysis and interpretation of data, or in the writing of the report. The authors and wider research team are independent from the funders. The authors had full access to all the data (including statistical reports and tables) in the study and take responsibility for the integrity of the data and the accuracy of the data analysis. The authors had the final responsibility for the decision to submit for publication.

## Data sharing

Technical appendix, statistical code, and dataset available from the corresponding author (theresa.marteau@kcl.ac.uk).

## APPENDIX 1: Questionnaire used in the study

### Section 1

1. For me, having the antenatal blood test to find out if I am a carrier for sickle cell or thalassaemia would be:
2. For me, having the antenatal blood test to find out if I am a carrier for sickle cell or thalassaemia would be:
3. For me, having the antenatal blood test to find out if I am a carrier for sickle cell or thalassaemia would be:
4. For me, having the antenatal blood test to find out if I am a carrier for sickle cell or thalassaemia would be:
  Note: Please check that you have circled a number for EACH of the above four questions.
5. Antenatal screening for sickle cell and thalassaemia is a blood test that…
  (tick ✓ only 1 answer to this question)
6. How likely do you think it is that your baby might be affected by sickle cell or thalassaemia
  (tick ✓ only 1 answer to this question)
7. A baby can be affected by sickle cell or thalassaemia if…
  (tick ✓ only 1 answer to this question)
8. *Sickle cell anaemia* is
  (tick ✓ only one answer to this question)
9. *Thalassaemia major* is
  (tick ✓ only one answer to this question)
10. People with *sickle cell anaemia*
  (you can tick ✓ more than one answer to this question)
11. People with *thalassaemia major*
  (you can tick ✓ more than one answer to this question)
12. Genes for *sickle cell disorders* are more common in people whose family origins are:
  (you can tick ✓ more than one option)
13. Genes for *thalassaemia disorders* are more common in people whose family origins are:
  (you can tick ✓ more than one option)
14. If your results show that you have inherited a gene for sickle cell or thalassaemia, how likely do you think it is that your baby will be affected?
  (tick ✓ only 1 answer to this question)
15. Some women are offered special tests after they have been screened for sickle cell or thalassaemia. These tests are called amniocentesis and chorionic villus sampling (CVS).
16. If the special tests show that the baby has sickle cell or thalassaemia, the parents are offered counselling. The options available include:
  (you can tick ✓ more than one answer)
